# Channel activity of SARS-CoV-2 viroporin ORF3a inhibited by adamantanes and phenolic plant metabolites

**DOI:** 10.1038/s41598-023-31764-9

**Published:** 2023-04-01

**Authors:** Marina Sherif Fam, Christine Adel Sedky, Nancy Osama Turky, Hans-Georg Breitinger, Ulrike Breitinger

**Affiliations:** Department of Biochemistry, German University in Cairo, Main Entrance of Al Tagamoa Al Khames, New Cairo, New Cairo 11835 Egypt

**Keywords:** Ion channels, Pharmacology, Drug development

## Abstract

SARS-CoV-2 has been responsible for the major worldwide pandemic of COVID-19. Despite the enormous success of vaccination campaigns, virus infections are still prevalent and effective antiviral therapies are urgently needed. Viroporins are essential for virus replication and release, and are thus promising therapeutic targets. Here, we studied the expression and function of recombinant ORF3a viroporin of SARS-CoV-2 using a combination of cell viability assays and patch-clamp electrophysiology. ORF3a was expressed in HEK293 cells and transport to the plasma membrane verified by a dot blot assay. Incorporation of a membrane-directing signal peptide increased plasma membrane expression. Cell viability tests were carried out to measure cell damage associated with ORF3a activity, and voltage-clamp recordings verified its channel activity. The classical viroporin inhibitors amantadine and rimantadine inhibited ORF3a channels. A series of ten flavonoids and polyphenolics were studied. Kaempferol, quercetin, epigallocatechin gallate, nobiletin, resveratrol and curcumin were ORF3a inhibitors, with IC_50_ values ranging between 1 and 6 µM, while 6-gingerol, apigenin, naringenin and genistein were inactive. For flavonoids, inhibitory activity could be related to the pattern of OH groups on the chromone ring system. Thus, the ORF3a viroporin of SARS-CoV-2 may indeed be a promising target for antiviral drugs.

## Introduction

Coronaviruses (CoVs) belong to the order *Nidovirales*, family *Coronaviridae*, and subfamily *Coronavirinae*^1^. They are subdivided into four different genera named α-, β-, γ-, and δ-CoVs^2^. Coronaviruses have been known to infect humans^2–4^, usually causing mild respiratory infections such as a common cold. However, in the past 20 years, two major outbreaks occurred due to crossover of animal β- coronavirus to humans^5^. In 2002–03 humans were infected by bat coronavirus resulting in severe acute respiratory syndrome coronavirus (SARS-CoV) and in 2019, a novel coronavirus of bat origin that had spread to humans, had been discovered in Wuhan, China^6^. This new virus, named SARS-CoV-2, is a member of the β-coronavirus family and is responsible for the ongoing pandemic of COVID-19^1,7,8^.

SARS-CoVs are enveloped, positive sense single-stranded RNA viruses, with a genome of approximately 30 kb arranged into 14 open reading frames (ORF) encoding 31 proteins^8–11^. Spike (S), envelope (E), membrane (M) and nucleoprotein (N) are the four structural proteins forming the virus capsid. The S protein binds to the host receptor through the receptor-binding domain in the S1 subunit, while S2 subunit is responsible for membrane fusion^8^. The E protein belongs to the class of viroporins, integral membrane proteins functioning as ion channels and promoting virus release. It was found to be expressed in the ER and the Golgi apparatus forming an ion channel allowing the efflux of cations Na^+^, K^+^ and Ca^2+^, and is required for pathogenesis and stimulation of inflammation^8,12–15^. The E-protein of SARS-CoV has been characterized thoroughly, showing that its ion channel activity, as well as its interaction with cellular and other virus proteins is essential for virus replication^16–23^. The M protein is the most abundant structural protein in the viral envelope, the N protein wraps around the RNA forming the nucleocapsid^8^. Proteins from open reading frame 1, ORF1a and ORF1b, known as the non-structural proteins (NSPs), form the intracellular replicase-transcriptase complex^24^. In addition to the structural proteins and NSPs, coronaviruses encode several accessory proteins which vary in number, location and size in the different viral subgroups, and are thought to contain additional functions that are often not required for virus replication, but are involved in pathogenicity in the natural host^25^.

**Figure 1:**
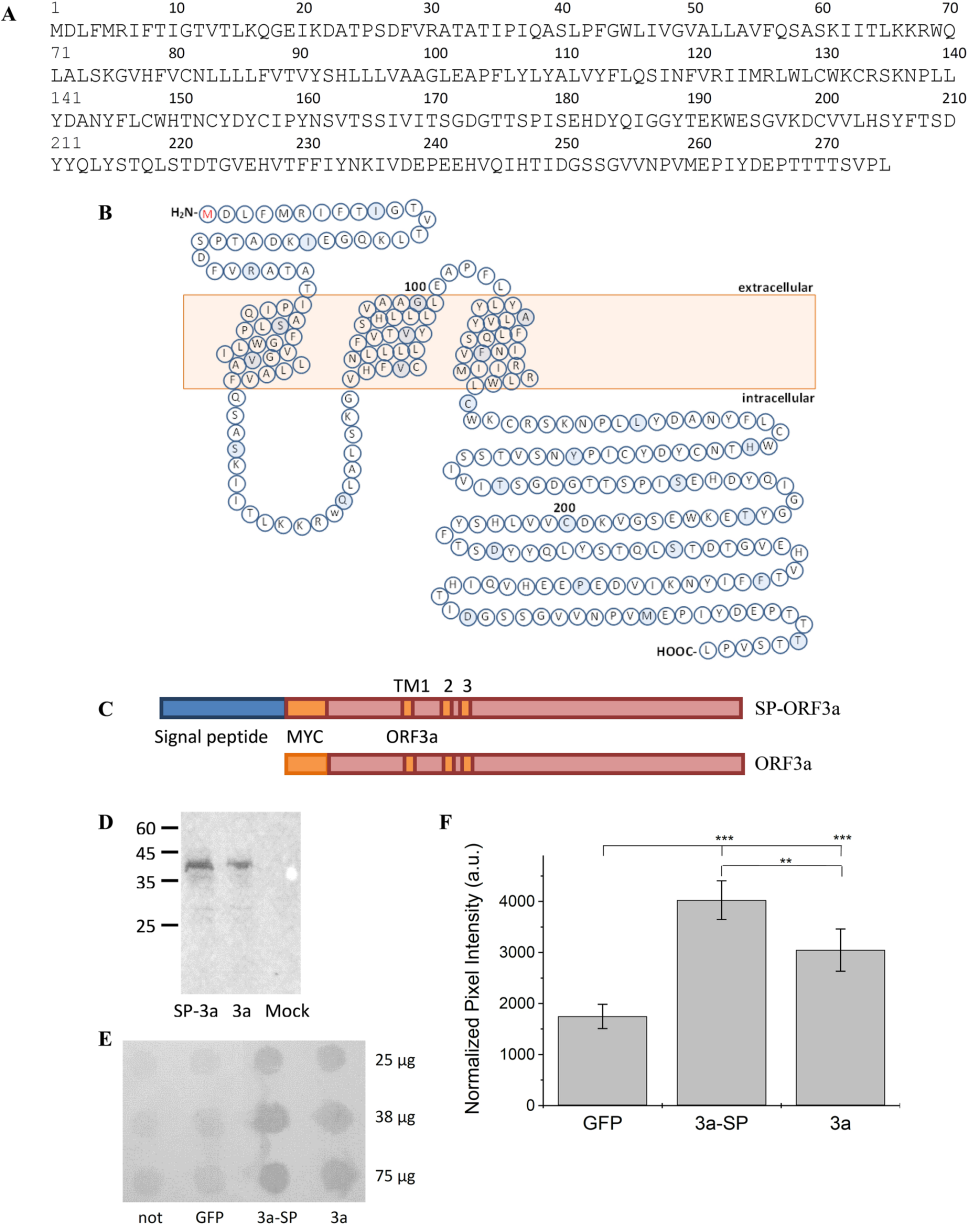
Sequence, topology and recombinant expression of ORF3a constructs. (**A**) Sequence of the ORF3a protein (GenBank NC_045512.2). (**B**) TM topology and amino acid distribution of ORF3a. Positions 100 and 200 are labelled. Every 10th amino acid is coloured. (**C**) Scheme of SP-MYC-ORF3a and MYC-ORF3a. (**D**) Western blot confirming the expression of ORF3a SARS-CoV-2 in HEK293 cells. Molecular weights of markers are indicated. Lane 1: HEK293 cells transfected with DNA encoding SP-ORF3a; lane 2: ORF3a without signal peptide; lane 3: mock-transfected HEK293 cells (GFP). Theoretical sizes of SP-ORF3a and ORF3a are 39.8 kDa and 35.6 kDa. Note that both constructs show an immune signal of the same size, indicating efficient cleavage of the signal peptide. One complete blot is shown. (**E**) Surface Expression Analysis: HEK293 cells were transfected with cDNA encoding ORF3a protein (3a), and ORF3a protein fused to a membrane-directing signal peptide (3a–SP), untransfected (not) and mock-transfected cells were used as controls (GFP); 25, 37.5 and 75 µg of total protein were applied per spot. One complete dot blot from a representative experiment is shown. (**F**) Quantification of Blot intensities. Two independent experiments were performed and different concentrations of total protein spotted (n = 5). Spot intensities were quantified using ImageJ software, intensities of each spot summated and normalized to the total intensity count of each row. Average ± standard deviation from all determinations is shown, p-values are indicated, ***: *p* < 0.001, **: *p* < 0.005 (one-way ANOVA).

ORF3a (Fig. 1A, B) belongs among the group of viroporins. It is the largest accessory protein (275 amino acids) of SARS-CoV-2 and shares 72.7% identity with the ORF3a protein of SARS-CoV^9,12^. Despite this high degree of homology, ORF3a was classified as unique to SARS-CoV-2^24,26^, since it was found to be structurally and functionally different from its counterpart in SARS-CoV. The importance of the viroporins of SARS-CoV for virus replication has been studied in detail^27^, showing that activity of both, E- and ORF3a protein is required for full virus replication and virulence. Simultaneous deletion of both viroporins renders the virus incapable of replication. Deletion of either E- or ORF3a protein resulted in viruses that were viable, but showed reduced replication rate and in vivo toxicity in a mouse model^27^. While the contribution of the E-protein to virulence of SARS-CoV is stronger, presence of functional ORF3a is nevertheless essential for full activity of the virus. ORF3a is the only viroporin that comprises three transmembrane domains (Fig. 1B), with the N-terminus located at the luminal side of the ER and the C-terminal domain directed to the cytosol^28^. The ion channel function of ORF3a of SARS-CoV was shown to be unrelated to viral replication in one study^27^, but associated with pro-apoptotic activity that affected replication in another^29^. Notably, different animal models were used in these studies. ORF3a activity triggered secretion of apoptotic mediators IL-1beta and NF-κB^30,31^, and increased production of cellular chemokines^32^. The cytoplasmic domain of ORF3a was reported to be involved in the induction of pro-apoptotic reactions by activating the mitochondrial p38 MAP kinase death pathway, which could be repressed by a p38 MAPK inhibitor^33^. Further, ORF3a was shown to induce intracellular membrane rearrangement^34^. Ion selectivity investigations on ORF3a channels reconstituted in proteoliposomes revealed nonselective cation channel activity with a permeability order of Ca^2^^+^  > K^+^  > Na^+^  > NMDG^+^^35^. ORF3a has been suggested as target for antiviral therapy, and kaempferol and related flavonoids were shown to inhibit the ORF3a channel in vitro^36^. The anthraquinone derivative emodin is an inhibitor of ORF3a of SARS-CoV, and the related beta-coronavirus HCoV-OC43, and was indeed able to repress virus release from the latter^37^.

ORF3a of SARS-CoV-2 was shown to prevent autophagy, one of the major defence mechanisms of cells in the fight against pathogens^38–41^. ORF3a prevents fusion of autophagosomes with lysosomes by blocking the interaction of the homotypic fusion and protein sorting (HOPS) complex with the SNARE complex and RAB7^38^. SARS-CoV-2 ORF3a activates the NLRP3 inflammasome^42^, and has pro-apoptotic activity, reported to be weaker than in case of SARS-CoV^43^. This reduced apoptotic activity was speculated to account – at least partially – for the lower virulence of SARS-CoV-2 compared to SARS-CoV. Reduced apoptotic reaction may lead to an overall reduced defensive response, allowing the virus to spread more widely into tissue even in case of mild clinical symptoms^43^. ORF3a of SARS-CoV-2 was shown to downregulate surface expression of MHC-1, thereby preventing detection and destruction of infected cells by CD8(+) T lymphocytes^44^. ORF3a of SARS-CoV and SARS-CoV-2 interacts with caveolin^45,46^.

Expression of the ORF3a protein in several in vitro systems describes its localization mainly in the Golgi region^46^ but also in the ER^31^, late endosomes^38^, lysosomes^47^, and the trans-Golgi network^47^. In case of SARS-CoV-2, localization of ORF3a in the plasma membrane, as well as in cytosolic compartments has been shown^43^. Thus, ORF3a is an essential contributor to infection and propagation of SARS-CoV-2, and its structure and function are of ongoing interest^26,48–52^. Indeed, viroporin inhibitors^53^ as well as potential inhibitors of ORF3a have been tested in vivo and in vitro^36,37^, and searched through in-silico approaches^54^, and potent inhibitors of ORF3a are of interest as antiviral drug candidates.

Given the essential role of viroporins in the virus life cycle, viroporin inhibitors are promising potential antiviral agents^12^. Classical viroporin inhibitors including amantadine and rimantadine have shown activity against numerous viral channels^53,55–61^. Another interesting substance class would be phenolics, naturally occurring secondary metabolites found in many plant tissues. They are known for powerful antioxidant, anti-inflammatory, immunomodulatory, and antibacterial activity, as well as for their action against various viruses^62^. Their antiviral activity involves interference with viral life cycle and pathogenesis^63^. An additional advantage of many polyphenols is the fact that their beneficial activity in humans is well established, and they can thus easily be repurposed as antiviral therapeutics^64,65^. Thus, flavonoids and other phenolics may serve as antiviral agents directly, or as lead structures towards the discovery of novel, specific viroporin inhibitors. In this study, we tested seven flavonoids – kaempferol, quercetin, epigallocatechin gallate (ECGC), nobiletin, apigenin, genistein, and naringenin. Given their reported antiviral activity, the phenolics resveratrol^66–69^, curcumin^70–73^, as well as 6-gingerol^74,75^, a component of ginger rhizome^76–78^ were also included in this study.

We show the inhibition of SARS-CoV-2 ORF3a activity by the classic viroporin inhibitors amantadine and rimantadine and by a series of ten flavonoids and phenolics. Toxicity of recombinantly expressed ORF3a channels was assessed using a cell viability assay, and could be correlated to inhibition of ion channel activity of recombinant ORF3a, measured by patch-clamp electrophysiology. Some of the tested compounds, including kaempferol, quercetin, EGCG, nobiletin, resveratrol and curcumin inhibited ORF3a activity, with IC_50_ values ranging between 1 and 6 µM, indicating that ORF3a may be a target for antiviral therapy.

## Materials and methods

### ORF3a DNA constructs

SARS-CoV-2 ORF3a cDNA (gift from Prof. Jun Wang, Rutgers University, NJ, USA), corresponding to the Severe acute respiratory syndrome coronavirus 2 isolate Wuhan-Hu-1 (GenBank NC_045512.2) (Fig. 1A, B) was cloned into the pRK vector using BamHI and HindIII restriction sites. One construct included the myc-tag (EQKLISEEDL) for verification in Western blotting and dot blot analysis. The second construct included the myc-tag and an additional signal peptide (SP), MWTPRVPPPRPALSFFLLLLLGVTYG, taken from the murine semaphorin-6B precursor (Fig. 1C).

### Cell culture and transfection

HEK293 cells (ATCC, LGC Standards GmbH, Wesel, Germany) were cultured in 10 cm tissue culture dishes in Dulbecco's Modified Eagle Medium, low glucose (DMEM, Sigma-Aldrich, Deisenhofen, Germany) supplemented with 10% FBS (Invitrogen, Karlsruhe, Germany) and Penicillin/ Streptomycin (Sigma-Aldrich, Deisenhofen, Germany) at 5% CO_2_ and 37 °C in a water-saturated atmosphere. For electrophysiological experiments, cells were plated on acetone treated glass coverslips in 24 well plates and transfected one day after passage using 1 µg of target cDNA, 1 µg of green fluorescence protein cDNA and 3 µl polyethyleneimine (PEI) (Sigma-Aldrich, Deisenhofen, Germany) per well. Measurements were performed 2–3 days after transfection.

### Membrane preparation

HEK293 cells were grown in 6 cm plates, transfected with 6 µg of cDNA and 20 µg of PEI as described under ‘Cell Culture and Transfection’. HEK293 cells were harvested 3 days after transfection using PBS (1.5 mM KH_2_PO_4_, 6.5 mM Na_2_HPO_4_, 3.0 mM KCl, 137 mM NaCl) and centrifuged at 2000 g. The pellet was resuspended in hypotonic buffer (20 mM K-Phosphate pH 7.4, 5 mM EDTA, 5 mM EGTA, 1 tablet cOmplete™ Mini Protease Inhibitor Cocktail (Sigma-Aldrich, Deisenhofen, Germany)) and kept on ice for 20 min. Cells were homogenized using a glass cell homogenizer and then centrifuged at 17,000 rpm for 20 min. Pellets were resuspended in hypotonic buffer and total protein concentration was measured prior to SDS PAGE.

### Western blot and dot blot analysis

For Western blotting, membrane suspension (30 µg of total protein) in SDS loading dye (2 × loading dye: 0.125 M Tris–HCl pH 6.8, 20% glycerol, 4% SDS, 0.02% bromophenol blue, 0.2 M DTT) were loaded per lane and subjected to SDS-PAGE. We used a combination of a myc-tag polyclonal primary antibody from rabbit and alkaline phosphatase (AP) – conjugated Goat Anti-Rabbit secondary antibody (Proteintech, Martinsried, Germany). The blot was visualized using the reaction of 0.03% nitro blue tetrazolium (NBT) and 0.02% 5-bromo-4-chloro-3-indolyl-phosphate (BCIP) (both Carl Roth, Karlsruhe, Germany) in substrate buffer (100 mM Tris–HCl, pH 9.5; 100 mM NaCl; 5 mM MgCl_2_).

For the dot blot assay, HEK293 cells were grown in 6 cm plates and transfected as described under “Membrane preparation”. Three days after transfection, culture medium was removed and cells washed with PBS, blocked with fresh full medium for 20 min, then primary antibody was added to the living cells and incubated for 2 h at 37 °C. Secondary antibody was applied for 1 h, cells were harvested, homogenized, protein concentration determined, 25–75 µg of total protein per well were spotted onto a nylon blotting membrane in a dot blot apparatus and immune signal detected. Antibodies and detection reagents were the same as in Western blotting. Pixel intensity of dots was quantified using ImageJ software without further correction of the raw data. For each series of applied protein, pixel intensities for each well were normalized to the total pixel intensity of all peaks of each treatment. The percent of total intensity of each well could then be calculated. Averages were taken from dot blots from two independent expressions using two or three different amounts of total protein (n = 5).

### Cell viability assay

120 µl of a HEK293 cell suspension were seeded into a 96-well plate at ~ 60,000 cells per well. Cells were transfected one day after plating. Next, 6.5 µl of inhibitor stock in DMEM were added to achieve final concentrations as required (usually between 1 and 100 µM). Four days after transfection, the MTT assay was performed as described before^79^. Briefly, culture medium was removed, cells were washed once with PBS, and 100 µl/well of a 0.5% solution of 3-(4,5-dimethyldiazol-2-yl)-2,5-diphenyltetrazolium bromide (MTT) in PBS was added. Cells were incubated at 37 °C for 2 h. Solution was carefully removed and 100 µl/well of dimethyl sulfoxide (DMSO) were added to dissolve formazan crystals. Absorbance was read in a Victor-3 plate reader (Perkin-Elmer, Berlin, Germany) at a wavelength of 595 nm. Controls included GFP transfected cells to verify efficient transfection and the addition of 200 mM KCl to untransfected cells to induce complete cell death; the 200 mM KCl readings were subtracted from all other data as background correction. Each experimental condition was tested on (i) ORF3a-transfected cells, and (ii) control cells transfected with empty pRK vector. For quantification, viability readings for each condition were averaged. The quotient $\frac{Viability(ORF3a-exopressing\;cells)}{Viability(pRK-transfected\;cells)}$ was calculated and presented in Fig. 3. Each assay was performed in quadruples, a minimum of two independent experiments per condition were carried out (n ≥ 8).

### Electrophysiological recordings and data analysis

HEK293 cells were plated on glass cover slides; transfected with cDNA constructs of ORF3a or mock and co-transfected with GFP, followed by incubation for 2–3 days to allow protein expression. Cover slides were transferred to a recording chamber, immersed in extracellular buffer and whole-cell currents recorded using a HEKA EPC 10 (NPI electronics, Tamm, Germany) patch-clamp amplifier. Recording pipettes (thin-walled borosilicate glass, 100 × 1.5 mm, 0.86 mm inner diameter, Science Products, Berlin, Germany) were pulled using a Sutter P-1 pipette puller (NPI electronics). For recordings of current–voltage ramps, we used symmetrical external and internal electrophysiology buffers. External buffer consisted of (in mM) 135 NaCl, 5.5 KCl, 2 CaCl_2_, 1.0 MgCl_2_ and 10 HEPES (pH adjusted to 7.2 with NaOH); the internal buffer was (in mM) 140 CsCl, 1.0 CaCl_2_, 2.0 MgCl_2_, 5.0 EGTA and 10 HEPES (pH adjusted to 7.2 with CsOH). The applied voltage ramp ranged from − 60 mV to + 50 mV in 10 mV steps. For measurements in presence of inhibitor, we added the inhibitor to the extracellular bath before patching cells and recording; a minimum of 6 cells per each concentration of each inhibitor were averaged. Dose–response curves were constructed from the analysis of current differences at − 60 mV, and IC_50_ values were determined using a non-linear fit to the equation I_obs_ = I_max_ / [1 + ([I]/IC_50_)].

## Results

### ORF3a constructs and expression of recombinant ORF3a protein

To investigate the ion channel properties of recombinant ORF3a we used a cDNA construct of ORF3a from the SARS-CoV-2 Wuhan wildtype, GenBank accession no NC_045512.2 (gift from Prof. Jun Wang, Rutgers U, NJ, USA) which was cloned into the mammalian expression vector pRK5. Since the main ion channel activity of viroporins is intracellular, related to ER membranes, the late endosome and lysosomes, we cloned ORF3a into the pRK5 vector including a myc-tag for detection in Western blotting (Fig. 1C). No signal peptide for plasma membrane transport was present in this construct which was used to show the effect of active ORF3a expression in cell viability assays on HEK293 cells. Electrophysiological measurements, on the other hand, require transport to the plasma membrane. Here, we introduced a membrane-directing N-terminal signal peptide from the murine semaphorin-6B precursor in addition to the myc-tag to the ORF3a (Fig. 1C). Recombinant expression of ORF3a was confirmed using HEK293 cells that were transfected with cDNA of SP-ORF3a and ORF3a, or a mock construct (GFP), and subjected to SDS PAGE and Western blot analysis three days after transfection (Fig. 1D). Specific signals were observed in Western blots for SP-ORF3a and ORF3a. Both proteins gave an immune signal of the same size, consistent with effective cleavage of the signal peptide during trafficking. We verified surface expression through dot blot analysis on native, living cells. To this end, HEK293 cells were transfected with target cDNA, allowed to express viroporin for three days, followed by application of primary and secondary antibodies directly to the living cells. Under these conditions, only plasma membrane-located ORF3a would be accessible to the antibody (Fig. 1E). When we compared dot blot intensities after subtracting the background signal (GFP), we found a ratio of ~ 1.75 : 1 for SP-ORF3a over ORF3a (Fig. 1F). Apparently, ORF3a – although mainly transported to intracellular membranes – was also found in the plasma membrane, even in absence of a signal peptide. Notably, addition of the semaphorin-6B signal sequence increased the transport to the plasma membrane significantly (Fig. 1F).

### Activity of the ORF3a viroporin – cell-based assays

**Figure 2:**
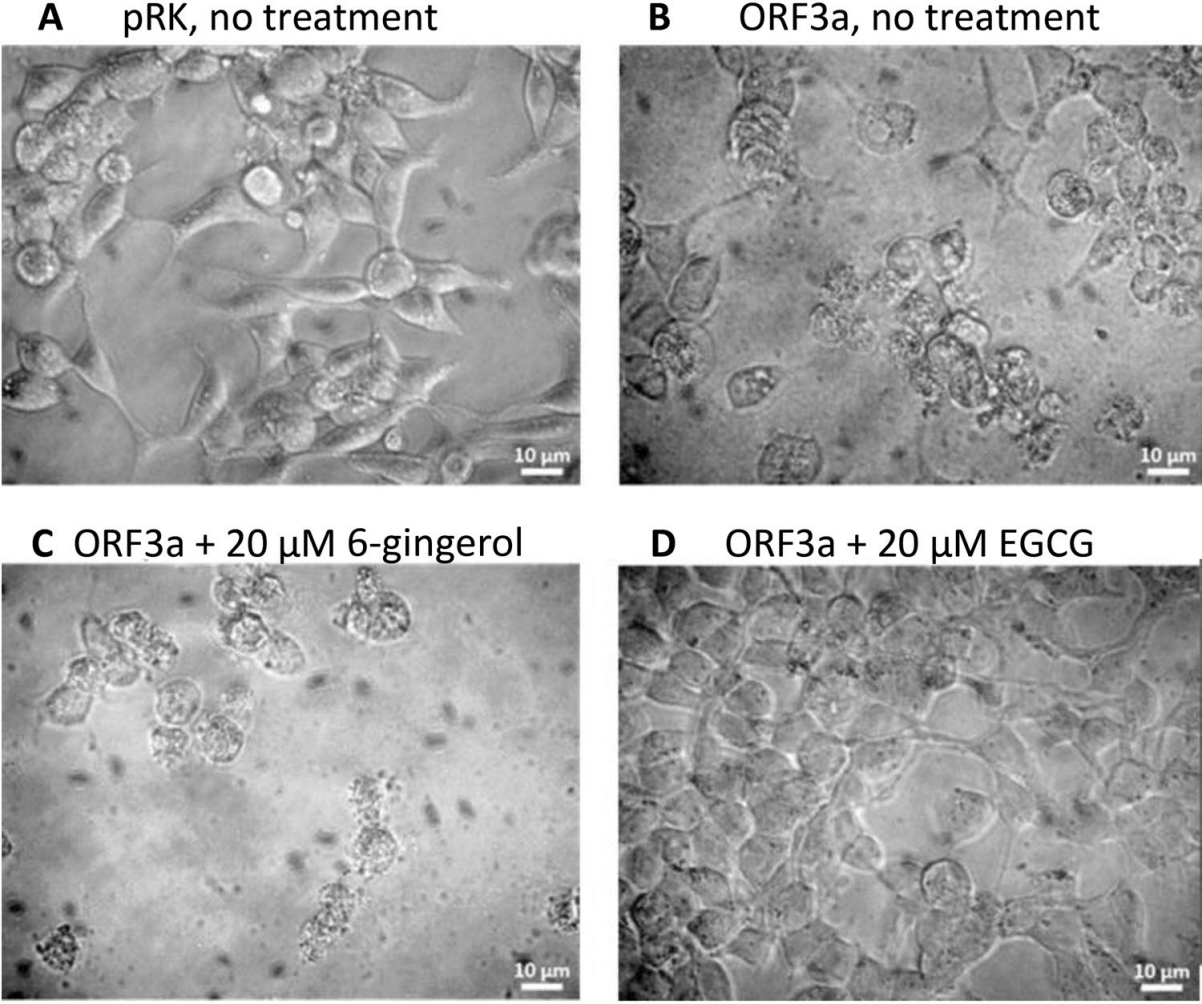
Morphology of HEK293 cells in absence and presence of ORF3a inhibitors. (**A**) HEK293 cells transfected with empty pRK vector. (**B**–**C**): HEK293 cells transfected with ORF3a. (**B**) untreated; (**C**) treated with 6-gingerol (inactive against ORF3a); (**D**) treated with EGCG (active ORF3a inhibitor).

Viroporin activity of ORF3a was analyzed using a cell viability assay system that we had previously established for the viroporin channels of hepatitis (p7), and the E protein of SARS-CoV^79,80^. Intracellular activity of viroporin channels damages and eventually destroys cells, leading to reduced overall viability of the culture. Treatment of ORF-3a-transfected cells with viroporin inhibitors should restore cell viability^79,80^. Indeed, morphology and viability/survival of ORF3a transfected cells changed compared to control. Four days after transfection, mock transfected cells looked healthy with normal morphology (Fig. 2A), while ORF3a transfected untreated cells were shrinking and fragmentation into membrane-bound apoptotic bodies could be observed (Fig. 2B). Cells treated with 6-gingerol (inactive against ORF3a) showed the morphology of dying cells (Fig. 2C), while treatment with epigallocatechin gallate (an active inhibitor of ORF3a) resulted in healthy cells with normal morphology (Fig. 2D). Thus, viroporin inhibitors were able to prevent viroporin-induced damage to transfected cells.

**Figure 3:**
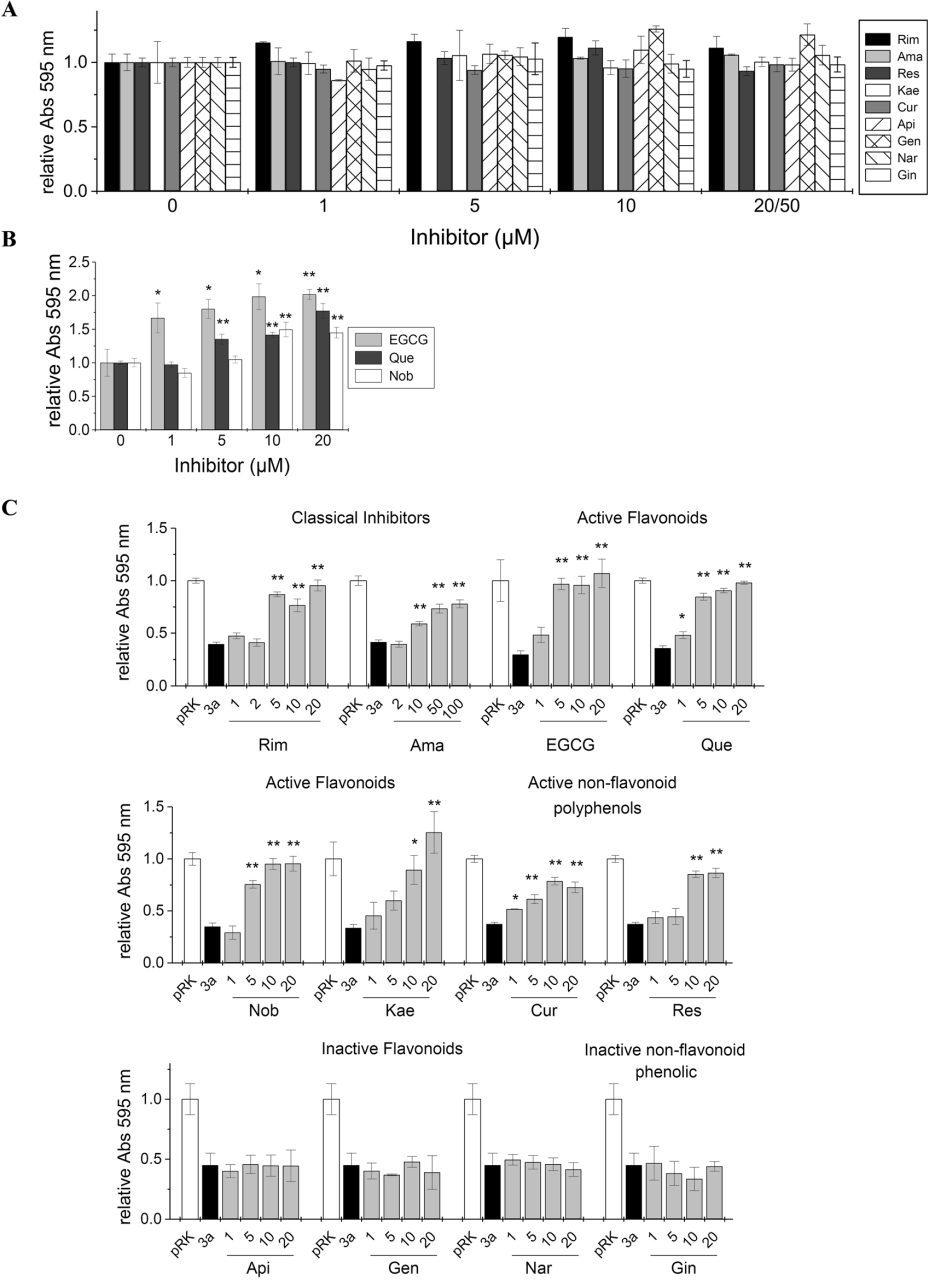
MTT cell viability assay of ORF3a transfected cells to investigate inhibitory action on ORF3a. (**A**) HEK293 cells were transfected with empty vector (control) and treated with the indicated inhibitors. Compounds did not affect cell viability. (**B**) Quercetin, EGCG and nobiletin at concentrations of 5 µM (Que), 10–20 µM (EGCG, Que and Nob) led to increased cell viability, indicating proliferative activity. (**C**) Viability test of viroporin inhibitors candidates. HEK293 cells were transfected with ORF3a and treated with increasing concentrations of inhibitor. For each data set, the first bar represents the control value (= 1) (white), then relative cell viability after expression ORF3a (black), followed by normalized viability readings at increasing concentrations of inhibitor (grey). (**A**–**C**) Significance relative to ORF3a-transfected cells in absence of inhibitor was determined using one-way ANOVA with * = *p* < 0.05; ** = *p* < 0.01, no sign = not significant. Abbreviations are as follows: Rim = rimantadine, Ama = amantadine, EGCG = epigallocatechin gallate, Que = quercetin, Nob = nobiletin, Res = resveratrol, Kae = kaempferol, Cur = curcumin, Api = apigenin, Gen = genistein, Nar = naringenin, Gin = 6-gingerol.

**Figure 4:**
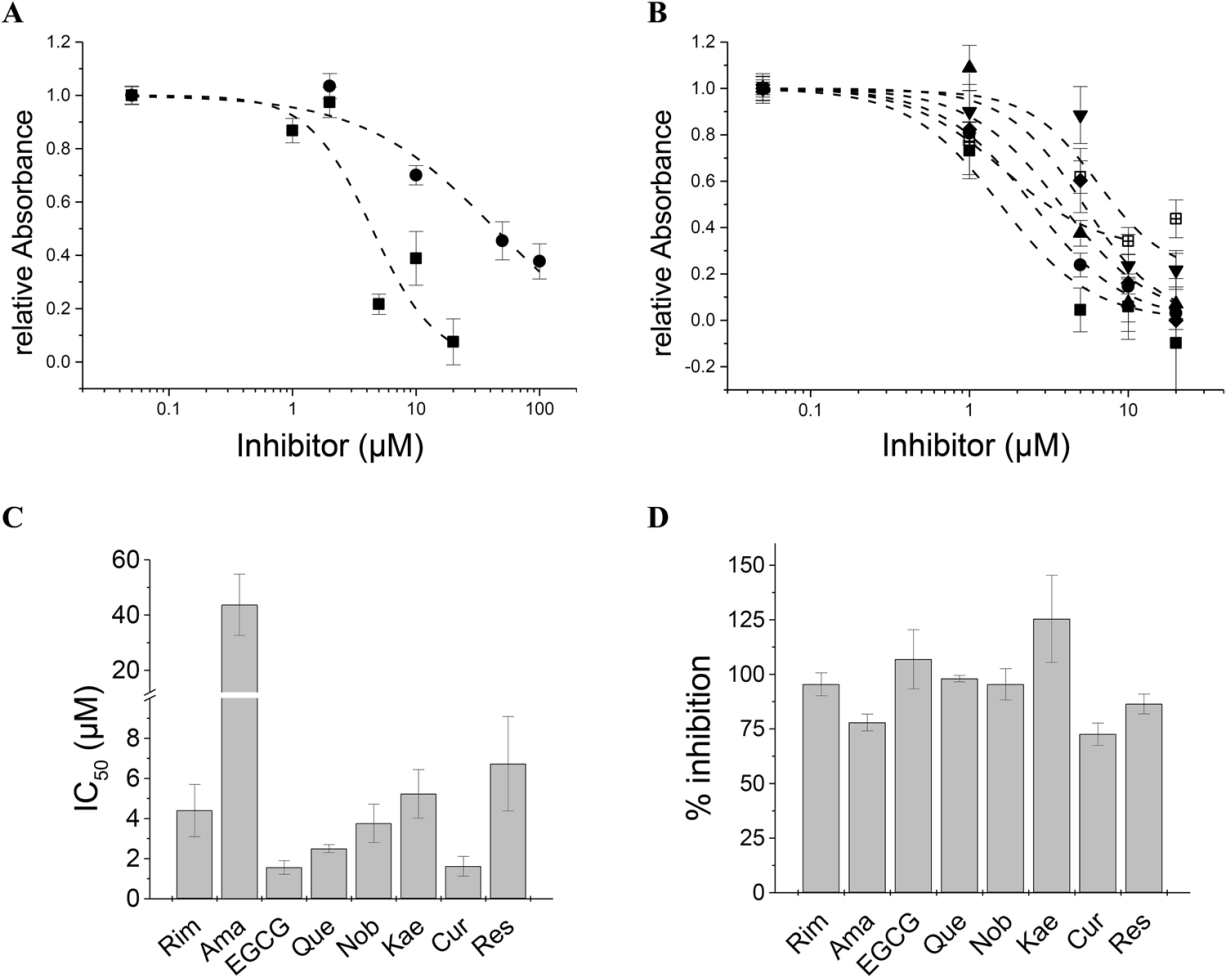
Concentration–response analysis of viroporin inhibition. (**A**, **B**) Concentration–response curves. ORF3a activity was calculated from viability data (see Methods), and normalized to viability data in absence of inhibitor. Note that in cases, where cell viability of treated ORF3a expressing HEK293 cells was increased as compared to control, relative absorbances could reach values below 0 in the IC_50_ graph. (**A**) Classical viroporin inhibitors; solid squares = rimantadine, solid circles = amantadine (**B**) Test compounds; solid squares = EGCG, solid circles = quercetin, up triangles = nobiletin, down triangles = resveratrol, diamonds = kaempferol, crossed squares = curcumin. (**C**) IC_50_ values of classical inhibitors and flavonoids. Abbreviations are as in Fig. 3. (**D**) Percentage inhibition was obtained from MTT data at the highest inhibitor concentration. All active inhibitors achieved complete inhibition (~ 100%) except amantadine (78%), curcumin (72%) and resveratrol (86%).

We then used an MTT-based cell viability assay to quantify ORF3a activity and the action of inhibitors and candidate substances on the ORF-3a channel. Inhibitors were applied to ORF-3a-, or mock transfected cells at the same concentrations. In this way, cytotoxic or proliferative effects of the inhibitors could be assessed. Most inhibitors had no effect on cell viability (Fig. 3A). Exceptions were EGCG, quercetin and nobiletin, where concentrations of 5 µM and higher (> 1 µM for ECGC) led to increased cell proliferation as compared to untreated controls (Fig. 3B). We analysed two classical viroporin inhibitors rimantadine (Rim) and amantadine (Ama), a series of seven flavonoids as well as the non-flavonoid phenolics resveratrol (Res), curcumin (Cur), and 6-gingerol (Gin) (Fig. 3C). Rimantadine and amantadine were active inhibitors with IC_50_ values of 4.4 ± 1.3 µM, and 43.7 ± 11.1 µM, respectively (Fig. 4A, C). EGCG, quercetin (Que), nobiletin (Nob), kaempferol (Kae) and curcumin (Cur) were inhibitors of ORF3a, reversing viroporin-induced cell damage with IC_50_ values (in µM) of 1.6 ± 0.3 (EGCG), 2.5 ± 0.2 (Que), 3.8 ± 0.9 (Nob), 5.2 ± 1.2 (Kae) and 1.6 ± 0.5 (Cur) (Fig. 4B, C). Resveratrol was an active inhibitor with an IC_50_ of 6.7 ± 2.3 µM. Most inhibitors showed complete (100%) inhibition of ORF3a activity at 20 µM concentration. Exceptions were amantadine, curcumin and resveratrol with maximal inhibition of 77.9 ± 3.9 µM (Ama), 72.6 ± 5.1 (Cur) and 86.5 ± 4.5 (Res) (Fig. 4D). We found apigenin (Api), genistein (Gen) and naringenin (Nar), as well as 6-gingerol (Gin) to be inactive against ORF3a (Fig. 3C).

### Activity and inhibition of ORF3a – patch-clamp electrophysiology

**Figure 5:**
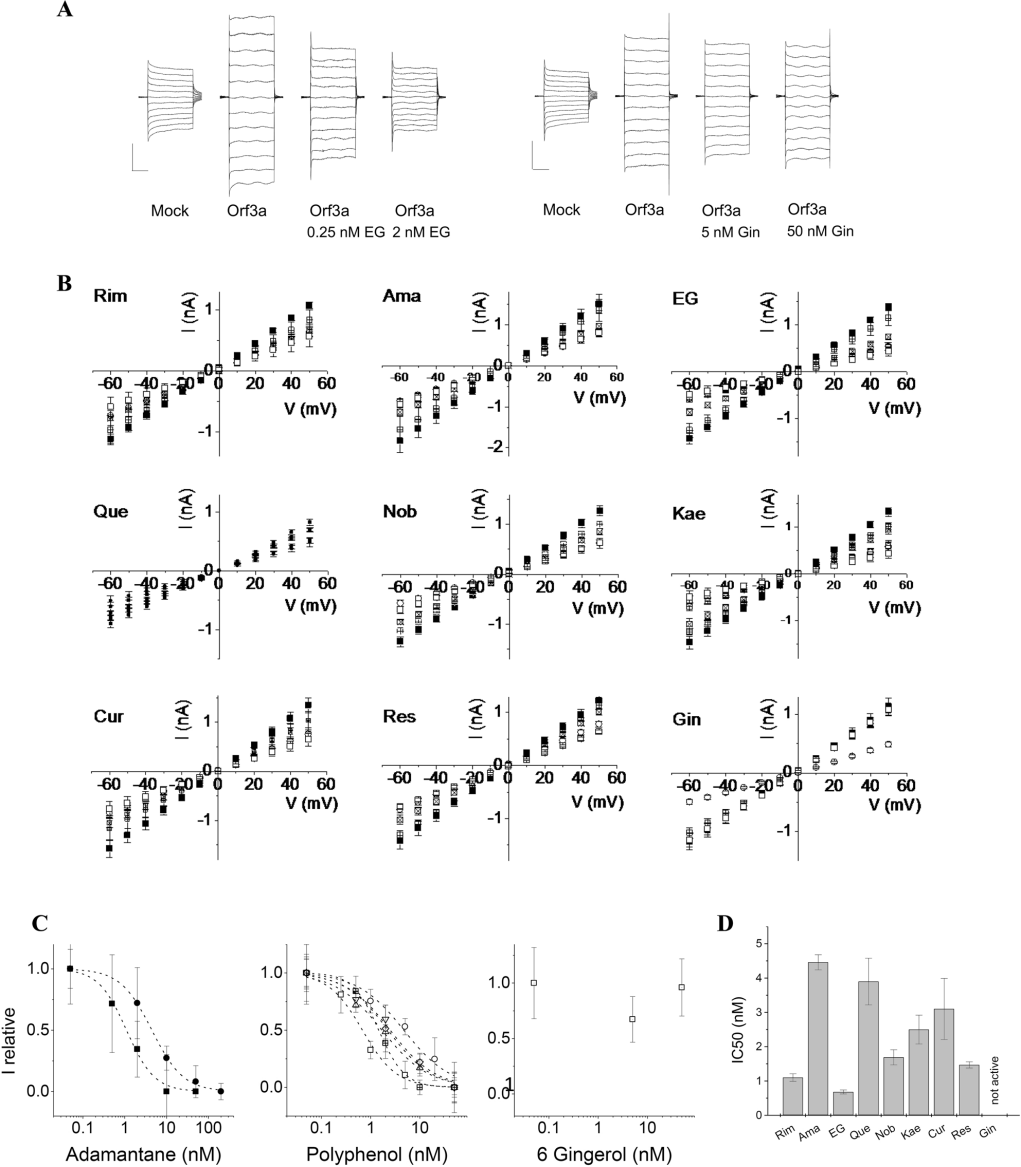
Patch-Clamp Electrophysiology measurements from HEK293 cells expressing SP-ORF3a. (**A**) IV ramp form − 60 mV to 50 mV was applied to mock-transfected cells and compared to IV ramps from cell transfected with SP-ORF3a in the absence and presence of inhibitors. Currents from ORF3a-expressing cells were increased compared to mock-transfected control cells. Currents were reduced and reached control values upon treatment with classical inhibitors (rimantadine and amantadine) or phenolics. Vertical and horizontal scale bars denote 0.5 nA and 10 ms, respectively. EG = epigallocatechin gallate. (**B**) Current–voltage relationship of all tested inhibitors. Open circles = mock transfected HEK293 cells; squares = ORF3a expressing cells without inhibitor; crossed squares = small inhibitor concentration (0.5–1 nM), crossed square = intermediate inhibitor concentration (1–5 nM); open squares = highest inhibitor concentration (20–50 nM). (**C**) IC_50_ curves from patch-clamp inhibition data: solid squares = rimantadine, solid circles = amantadine, open squares = EGCG, open circles = quercetin, up-triangles = nobiletin, down-triangles = kaempferol, diamonds = curcumin, crossed squares = resveratrol, dotted squares = 6-gingerol. (**D**) Summary of IC_50_ values from patch-clamp electrophysiology. Abbreviations are as listed in Fig. 3.

To correlate cell viability data to ion channel activity of ORF3a, we performed patch-clamp electrophysiology for all compounds that showed activity and for one inactive compound, namely 6-gingerol. Mock- or ORF3a-transfected cells were voltage-clamped and current–voltage (IV) ramps in the range from − 60 to + 50 mV were recorded. Presence of active ORF3a resulted in increased currents compared to control cells, due to viroporin channel activity on the cell surface. Currents from 5–6 cells for each condition were averaged. Addition of inhibitor reduced the currents in the IV ramp to control levels in a concentration-dependent manner. Mock transfected cells at − 60 mV gave currents in the range of 0.5 to 0.8 nA, which increased to 1.2–1.5 nA for ORF3a transfected cells. Data sets in presence of inhibitors and corresponding controls were always recorded on the same day and compared (Fig. 5A, B). Results from patch-clamp measurements showed similar trends as those from MTT results: activity of amantadine + (IC_50_ = 4.5 ± 0.2 nM) was approximately fourfold lower than that of rimantadine (IC_50_ = 1.1 ± 0.1 nM). IC_50_ values were 0.7 ± 0.1 nM for EGCG, 3.9 ± 0.7 for quercetin, 1.8 ± 0.3 for nobiletin, 2.4 ± 0.5 for kaempferol, 3.1 ± 0.9 for curcumin, and 1.5 ± 0.1 for resveratrol, while 6-gingerol was inactive (Fig. 5C, D).

## Discussion

Since viroporins are involved in various steps of the virus life cycle including assembly, budding, envelope formation, virus release, and inflammasome activation, they represent a promising target for antiviral drugs ^15,59,80^. Amantadine and rimantadine are well-characterized viroporin inhibitors, known to be active against a number of viroporins including the influenza M2 ion channel^59,60,81^, p7 channels of the hepatitis C virus^58,82^, and E protein of SARS-CoV^61,80^. To ensure sufficient plasma membrane expression of ORF3a – an essential requirement for patch-clamp electrophysiology – we attached an N-terminal signal peptide derived from murine semaphorin 6B. Plasma membrane expression of ORF3a was verified by Western blot and dot blot analysis. Presence of a membrane-directing signal peptide increased the expression of ORF3a on the plasma membrane.

We determined the activity of known viroporin inhibitors and a series of phenolic compounds against recombinant ORF3a protein using (i) a cell-based assay where viroporin-induced loss of cell viability and its restoration by viroporin inhibitors was quantified, and (ii) patch-clamp electrophysiology where the channel activity of viroporins can be observed directly. Reduced viability of cells expressing the viroporins p7 or E protein have been reported^79,80^. Both viroporins were shown to activate the NLRP3 inflammasome, in case of the E protein the transport of calcium ions was suggested as reason^83,84^.

Classical viroporin inhibitors were active against ORF3a. Inhibition was concentration dependent with the potency of rimantadine ~ tenfold and ~ fourfold higher than that of amantadine in cell viability assays and in patch clamp experiments, respectively. Higher potency of rimantadine over amantadine against viroporin channels has been reported before for the inhibition of p7 channels of HCV^79,85^. Both adamantanes showed inhibitory activity against the E viroporin of SARS-CoV in cell viability assays and patch-clamp measurements. Again, rimantadine was shown to be more potent than amantadine^80^. Higher antiviral activity of rimantadine in comparison to amantadine has also been reported for SARS-CoV-2 infected Vero E6 cells, where antiviral activity of rimantadine occurred primarily after viral entry rather than prior to viral entry^53^. Together, these results suggest that adamantane derivatives hold promising therapeutic potential against COVID-19.

**Figure 6:**
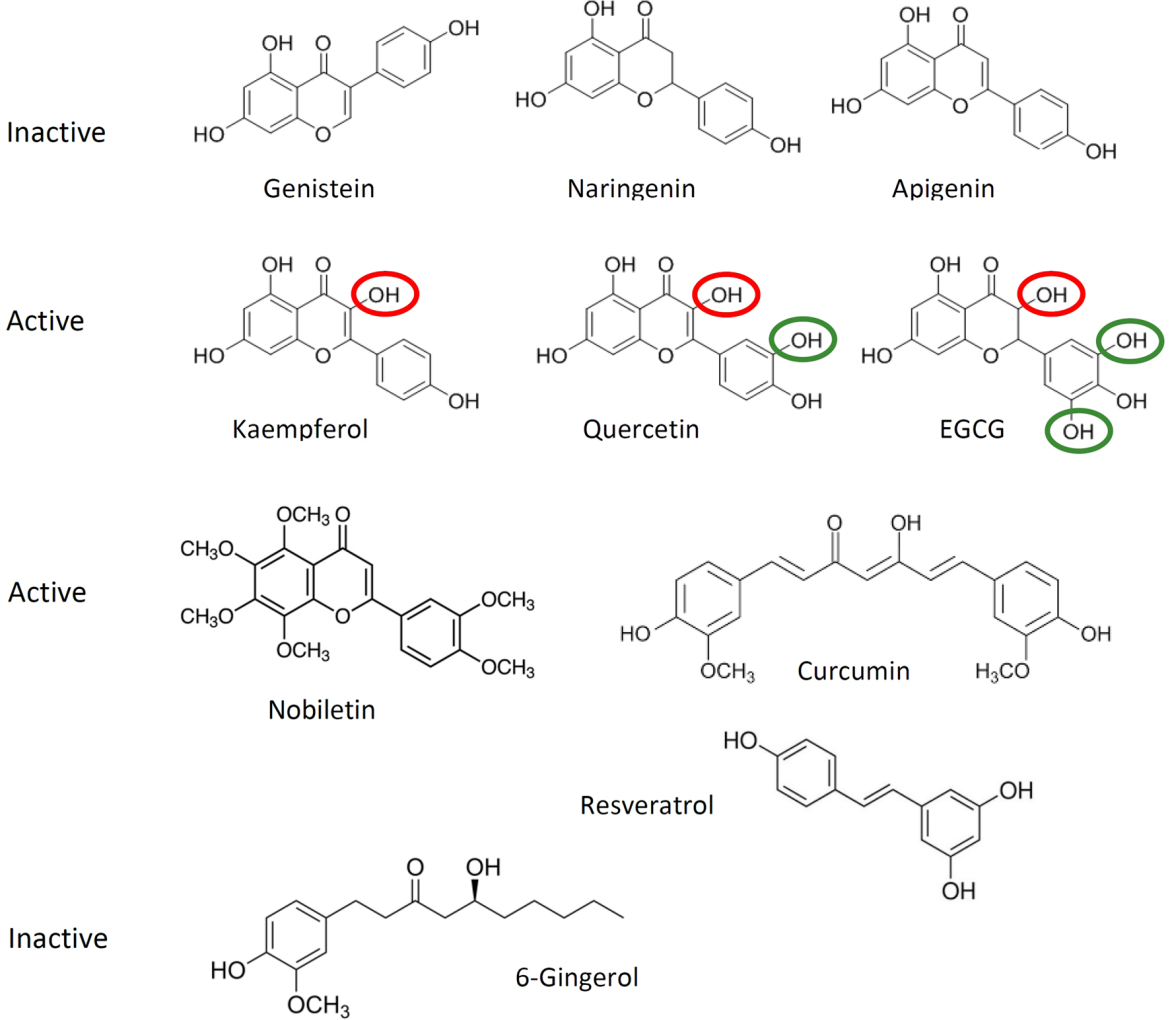
Structure–activity correlation of tested flavonoids and phenolics. Genistein, naringenin, and apigenin were inactive. Kaempferol, quercetin, EGCG, and nobiletin were active. The OH group encircled in red is essential for activity, those in green enhance activity. Note that nobiletin (no free OH groups), curcumin and resveratrol also inhibited ORF3a activity.

We tested ten phenolics – seven flavonoids, as well as the phenolics resveratrol, curcumin and 6-gingerol – for activity against ORF3a. Six of the tested phenolics (EGCG, quercetin, nobiletin, kaempferol, resveratrol, and curcumin) showed inhibitory activity against ORF3a-induced cytotoxicity, while four (genistein, naringenin, apigenin and 6-gingerol) were inactive. The activity of some natural phenolic compounds and inactivity of others indicates that the observed effects are not due to general antioxidant or metabolic effects of the compounds, but rather that some of these compounds can interact specifically with a distinct binding site on the ORF3a protein. The inactive flavonoids apigenin (flavone), naringenin (flavanone) and genistein (isoflavone) have three OH groups at positions C5 and C7 on ring A and C4’ on ring B (Fig. 6). These hydroxyl functions appear not to be relevant for interaction with ORF3a. In contrast, EGCG, quercetin and kaempferol (all flavonols) have the same basic chromone structure as apigenin, but carry an additional OH group on C3 of ring C (Fig. 6, red highlights). This hydroxy function seemed to be essential for activity against ORF3a, and its absence (as in apigenin) abolishes activity. Quercetin has one additional hydroxyl group on C3′ of ring B, EGCG has two additional hydroxyl groups at C3’ and C5’ of ring B (Fig. 6, highlighted in green). Presence of these hydroxyl groups – in addition to that in ring C – seem to enhance inhibitory potency against ORF3a further. The order of potency against ORF3a of kaempferol (5.23 ± 1.2 µM), quercetin (2.5 ± 0.2 µM) and EGCG (1.56 ± 0.34 µM) could be explained by the additional hydroxyl groups on ring B, where the increased number of available polar interactions or H-bridges appear to correlate with inhibitory potency. In contrast, partial saturation of the chromone skeleton between C2 and C3 (as seen in naringenin and EGCG) had no effect on activity. The role of OH groups on the flavonoid ring system has been studied with respect to antioxidant and protein binding activity^65,86,87^, but not yet correlated to antiviral effects.

Nobiletin showed potent activity against ORF3a. Although having the same chromone structure as the compounds discussed above, it lacks the essential C3-OH group on ring C, and all OH groups at C5, C6, C7, C8, C3′ and C4′ are methylated. Thus, nobiletin can only accept, but not donate OH bonds, and its polarity is severely reduced compared to the other tested flavonoids. The non-flavonoids, resveratrol (IC_50_ 6.73 ± 2.3 µM) and curcumin (IC_50_ 4.4 ± 1.7 µM) showed activity against ORF3a. In contrast, 6-gingerol was found inactive which would argue against a gingerol-binding site on ORF3a. Taken together, there appears to be a correlation between number and location of OH groups on the chromone ring system, but more structure–activity data are needed before a pharmacophore or a specific site of interaction can be delineated.

Current–voltage recordings confirmed the inhibitory activity for EGCG, quercetin, nobiletin, curcumin and resveratrol, although relative potency of quercetin was lower in the patch-clamp assay. 6-gingerol was also tested and showed to be inactive against ORF3a, as seen in cell viability tests. It was noted that IC_50_ values of viroporin inhibitors are different from those determined from cellular assays. This effect has been observed for p7 channels of hepatitis C^79,82,88^, as well as for the E protein of SARS-CoV^80^. This difference in apparent potency is likely due to availability of the protein to the inhibitor. In cellular assays, the inhibitor (applied extracellularly) has to cross the plasma membrane and may be sequestered or degraded before reaching its intracellular target, while in patch-clamp experiments the target viroporin is directly exposed to the inhibitor in the extracellular buffer.

Activity of plant phenolics against ORF3a has been reported in the literature: kaempferol and its glycosylated derivative juglanin inhibit ORF3a activity of SARS-CoV expressed in Xenopus oocytes, while naringenin and genistein do not^36^. The same study reported very little inhibition of ORF3a by quercetin^36^, as also observed here.

Antiviral activity of phenolics has been reported. Consistent with our observations, curcumin effectively inhibited viral replication in SARS-CoV infected Vero E6 cells^89^. In contrast, 6-gingerol was shown to have only low activity in the same expression system^90^. Quercetin showed potent anti-inflammatory activity and possible inhibition of the COVID-19-associated cytokine storm^91^. Clinical studies showed that the administration of nano-encapsulated curcumin to patients infected with COVID-19 resulted in a significant reduction in levels of IL-6 and IL-1β and improvement of patients’ symptoms^92^. Several in silico analyses suggest that curcumin, and some flavonoids may interact with SARS-CoV-2 proteins^24,93,94^. Thus, although some of the phenolics studied here (eg 6-gingerol, naringenin, apigenin, and genistein) were not active against ORF3a channels, they may yet possess activity against other proteins of SARS-CoV-2.

The antiviral activity of adamantane derivatives, classical viroporin inhibitors, and ten natural polyphenolics compounds was evaluated in HEK293 transfected with ORF3a of SARS-CoV-2 using cell viability assay and in patch-clamp analysis. Our findings revealed that the adamantane derivatives and six out of ten tested polyphenols have shown high potency against ORF3a and may indeed be promising medicinal agents. A structure–activity pattern for some flavonoids may emerge, which could lead to the identification of a distinct inhibitory site on the ORF3a protein, but further investigation is required. Flavonoids, phenolics and other natural compounds may be a promising source for novel antiviral agents.

## Supplementary Information

Supplementary Figure S1.

## Data Availability

The datasets used and/or analysed during the current study are available from the corresponding author on reasonable request.
