# Supplementary material for: Channel activity of SARS-CoV-2 viroporin ORF3a inhibited by adamantanes and phenolic plant metabolites
Source: Sci Rep. 2023 Apr 1;13:5328. doi: 10.1038/s41598-023-31764-9 (PMC10067842; doi:10.1038/s41598-023-31764-9)

The ORF3a viroporin of SARS-CoV-2 – Channel activity and inhibition by adamantanes and phenolic plant metabolites

Marina Sherif Fam, Christine Adel Sedky, Nancy Osama Turky, Hans-Georg Breitinger, Ulrike Breitinger*

Blots and Gels – original images

Figure 1 D – Western Blot of recombinant ORF3a

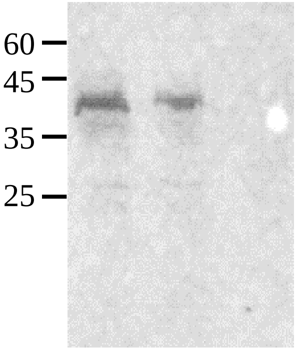

Complete membrane, loading scheme and chosen section
(E2 and some markers were not used in this study).

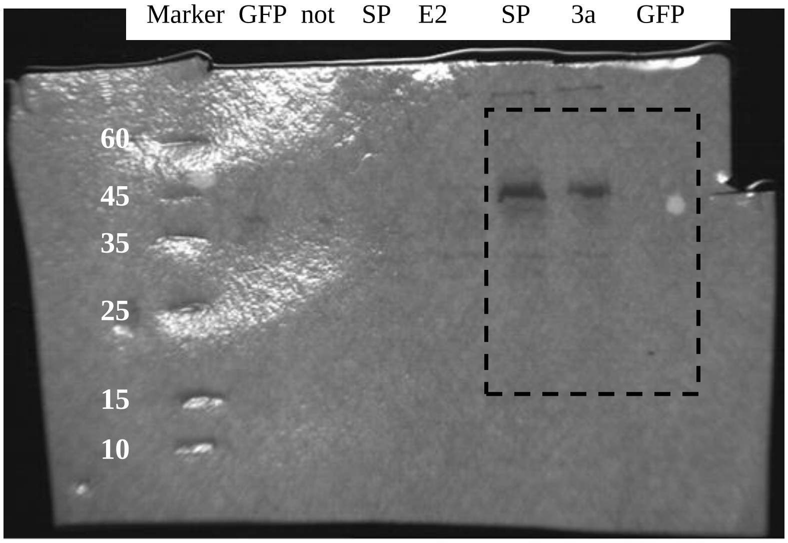

Figure 1 E – Dot Blot of plasma membrane-expressed ORF3a

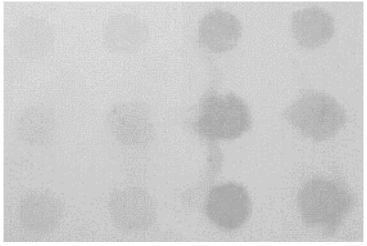

Complete membrane, loading scheme and chosen section
(3a of SARS-CoV was not used in this study).

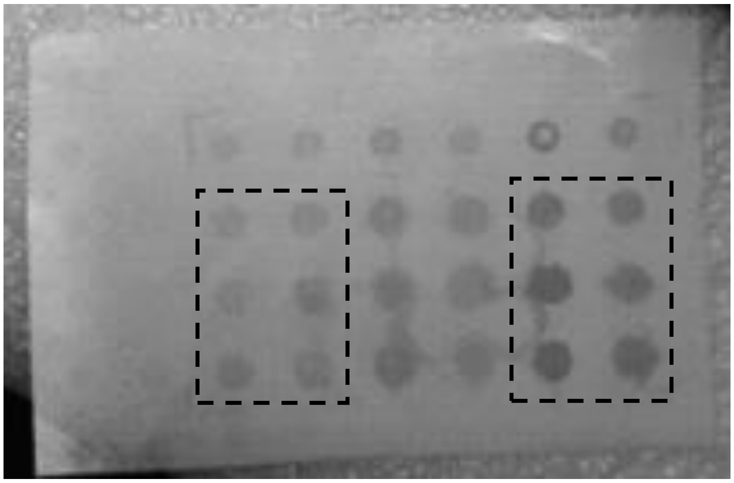

Supplement: Supplementary file 1 — Supplementary Figure S1. [file 41598_2023_31764_MOESM1_ESM.pdf]
